# Increased human Ca^2+^-activated Cl^-^ channel 1 expression and mucus overproduction in airway epithelia of smokers and chronic obstructive pulmonary disease patients

**DOI:** 10.1186/1465-9921-13-55

**Published:** 2012-06-25

**Authors:** Hiroki Iwashita, Keisaku Fujimoto, Shigeru Morita, Atsushi Nakanishi, Keishi Kubo

**Affiliations:** 1 Pharmaceutical Research Division, Takeda Pharmaceutical Company Limited, 2-26-1, Muraoka-higashi, Fujisawa, Kanagawa, 251-8555, Japan; 2 Department of Clinical Laboratory Sciences, Shinshu University School of Medicine, 3-1-1, Asahi, Matsumoto, Nagano, 390-8621, Japan; 3 The First Department of Internal Medicine, Shinshu University School of Medicine, 3-1-1, Asahi, Matsumoto, Nagano, 390-8621, Japan

**Keywords:** Ca^2+^-activated Cl^-^ channel 1 (CLCA1), Chronic obstructive pulmonary disease (COPD), Smoking, Mucus production, Airway epithelia

## Abstract

**Background:**

The mechanisms underlying the association between smoking and mucus overproduction remain unknown. Because of its involvement in other airway diseases, such as asthma, we hypothesized that Ca^2+^-activated Cl^-^ channel 1 (CLCA1) was associated with overproduction of mucus in the airways of smokers and COPD patients.

**Methods:**

Using real-time quantitative PCR analyses, we compared the CLCA1 mRNA expression levels in induced-sputum cells from COPD patients (n = 20), smokers without COPD (n = 5), and non-smokers (n =13). We also examined the relationship between CLCA1 protein expression and mucus production in lung airway epithelia of COPD patients (n = 6), smokers without COPD (n = 7), and non-smokers (n = 7).

**Results:**

CLCA1 mRNA expression was significantly up-regulated in the induced-sputum cells of COPD patients compared with cells of non-smokers (p = 0.02), but there was no significant difference compared with cells of smokers without COPD. Using immunostaining with an anti-CLCA1 antibody, semi-quantitative image analyses of airway epithelium demonstrated significantly increased CLCA1 expression in smokers without COPD (p = 0.02) and in COPD patients (p = 0.002) compared with non-smokers. There were significant negative correlations between CLCA1 protein expression and FEV_1_/FVC (r = −0.57, p = 0.01) and %predicted FEV_1_ (r = −0.56, p = 0.01). PAS staining for mucus showed that there was a significant positive correlation between CLCA1 protein expression and mucus production (r = 0.67, p = 0.001). These markers were significantly increased in smokers without COPD (p = 0.04) and in COPD patients (p = 0.003) compared with non-smokers (non-smokers < smokers ≤ COPD).

**Conclusions:**

CLCA1 expression is significantly related to mucus production in the airway epithelia of smokers and COPD patients, and may contribute to the development and pathogenesis of COPD by inducing mucus production.

## Background

COPD is a chronic inflammation of the airways, including the parenchyma and the pulmonary vasculature [1]. The pathological hallmarks of COPD are destruction of the lung parenchyma, which characterizes pulmonary emphysema, inflammation of the peripheral airways, and mucus hypersecretion. Goblet cell hyperplasia and mucus hypersecretion are prominent features of COPD, particularly during disease exacerbations. Mucus hypersecretion is associated with patient morbidity and with mortality among certain groups of patients.

Cigarette smoking is a major risk factor for COPD development; nearly 90% of COPD patients are smokers [2]. Cigarette smoke is a complex mixture of chemical compounds, including free radicals and other oxidants, which can potentially induce tissue damage, airway inflammation, goblet cell hyperplasia, and mucus overproduction [3,4]. However, the mechanisms underlying the association between smoking and mucus overproduction remain unknown.

Chloride channels are intimately associated with mucus secretion in the airways [5]. One of these channels, cystic fibrosis transmembrane conductance regulator (CFTR), is a well-known example in which defective mutations cause cystic fibrosis (CF) characterized by abnormal airway mucus [6-8]. CLCA is a newly identified family of chloride channel proteins that mediate Ca^2+^-activated Cl^-^ conductance. These channel proteins have 4 or 5 transmembrane regions and are 902 to 943 amino acids in length [9].

One of the more interesting features of this family is its wide distribution in human secretory organs [10]. The human CLCA1 gene is expressed in the digestive tract, including the colon, small intestine, stomach, and appendix. CLCA2 is mainly expressed in the trachea, while CLCA4 is mainly expressed in the colon and trachea. We previously found that mCLCA3 (mouse counterpart of CLCA1) was selectively expressed in airway goblet cells in a murine allergic asthma model and played a critical role in bronchial hyper-reactivity and mucus overproduction [11]. Further, only CLCA1 (originally designated CaCC1) was significantly up-regulated in the airway epithelium of bronchial asthma and played a direct role in mucus production in a human airway epithelial cell line [12].

In this study, to examine a role for CLCA1 in COPD and a mechanistic link between smoking and mucus overproduction, we compared the CLCA1 mRNA expression levels in induced-sputum cells from COPD patients, smokers without COPD, and non-smokers using real-time quantitative PCR analyses. We also examined the relationship between CLCA1 protein expression and mucus production in lung airway epithelia of COPD patients, smokers, and non-smokers.

## Methods

### Subjects and Sputum Collection and Analysis

The ethics committee of the Shinshu University School of Medicine approved this study (approval No. 255), and all subjects gave their informed consents. COPD was defined as a forced expiratory volume in 1 second (FEV_1_) to forced vital capacity (FVC) (FEV_1_/FVC) ratio of < 70%. Induced-sputum cells were obtained from 20 male patients with COPD (FEV_1_/FVC: 32.6-61.3%; %predicted FEV_1_: 32.0-123.6% after inhaling a beta-2 bronchodilator), 5 male smokers without COPD (FEV_1_/FVC: 81.0-97.5%), and 13 healthy non-smokers (FEV_1_/FVC: 80.8-96.1%) (Table 1). Patients with smoking-related COPD and without α1-antitrypsin deficiency were recruited from outpatient clinics. All COPD patients had a smoking history of ≥ 30 pack-years and no history of asthma or changes in symptoms. Based on the classifications for COPD severity outlined in the Global initiative for chronic Obstructive Lung Disease (GOLD) [1], 6 patients had mild COPD, 12 had moderate COPD, and 2 had severe COPD. All had been treated with bronchodilators including regular inhalation of an anti-cholinergic agent and/or beta-2 agonists and/or slow-releasing theophylline for more than 6 months prior to this study. The 5 smokers without COPD and the 13 normal healthy non-smokers were non-atopic and showed no abnormalities on pulmonary function test results.

**Table 1:** Subjects’ characteristics for CLCA1 mRNA expression in induced sputum

	**Non-smokers**	**Smokers without COPD**	**COPD**
Number of subjects (M / F)	13 (9 / 4)	5 (5 / 0)	20 (20 / 0)
Age (yrs)	29.8 ± 2.2	33.0 ± 4.9	72.3 ± 1.1 **††
Current / ex-smokers	-	5 / 0	4 / 16
Brinkman index (pack·years)	-	10.0 ± 2.4	52.1 ± 3.6
VC (%predicted)	99.0 ± 3.7	98.1 ± 2.2	97.8 ± 4.1
FEV_1_ (L)	3.5 ± 0.2	4.0 ± 0.3	1.4 ± 0.1 **††
FEV_1_ / FVC (%)	86.6 ± 1.5	88.4 ± 3.6	47.9 ± 2.0 **††
FEV_1_ (%predicted)	96.1 ± 3.3	101.7 ± 6.0	63.9 ± 5.7 **††

Results are means ± SEMs. Comparisons between groups were made by Student's t-tests (** p ≤ 0.01, compared with non-smokers; †† p ≤ 0.01, compared with smokers without COPD.).

Sputum was induced by inhalation of hypertonic saline as previously described [13]. Total cell counts, excluding squamous cells, were determined using a standard hemocytometer, normalized for mass, and expressed as cells x10^5^/g wet-weight sputum (Table 2). Cell smears were prepared with a cytocentrifuge (Autosmear, Sakura Seiki, Tokyo, Japan) and stained with May-Grünwald-Giemsa stain for differential cell counts; differentials were done in a blinded manner. Cellular total RNA was isolated using ISOGEN (Wako Pure Chemicals, Osaka, Japan), quantified by spectrophotometry, and reverse transcribed using a TaqMan Gold RT-PCR Kit (Applied Biosystems, Foster City, CA). cDNA samples were analyzed by real-time quantitative PCR using a Perkin-Elmer Applied Biosystems prism model 7700 sequence detection system, as previously described [12].

**Table 2:** Inflammatory cells in induced sputum

	**Non-smokers**	**Smokers without COPD**	**COPD**
Total cells (×10^5^cells/g)	36.6 ± 9.1	45.8 ± 17.5	39.4 ± 12.5
Macrophage (%)	35.5 ± 7.2	22.5 ± 11.2	8.3 ± 2.8 **
Lymphocyte (%)	2.9 ± 0.5	3.3 ± 0.9	3.8 ± 0.7
Neutrophil (%)	61.1 ± 7.3	73.8 ± 11.8	82.5 ± 4.0 **
Eosinophil (%)	0.5 ± 0.3	0.5 ± 0.5	5.5 ± 2.0 *

Results are means ± SEMs. Comparisons between groups were made by Student's t-tests (* p ≤ 0.05, ** p ≤ 0.01, compared with non-smokers).

### Specimens for Histochemical Analysis

Lung tissue specimens were obtained from lobectomies for lung cancer treatment. These were obtained from 6 male patients with mild to moderate COPD (FEV_1_/FVC: 57.3-69.5%; %predicted FEV_1_: 78.6-87.9% after inhaling a beta-2 bronchodilator), 7 smokers (FEV_1_/FVC: 70.2-85.1%), and 7 non-smokers (FEV_1_/FVC: 78.6-89.4%). All smokers and non-smokers did not show COPD or emphysematous changes based on high-resolution computed tomography (Table 3). All smoking-related COPD patients and without α1-antitrypsin deficiency exhibited irreversible airflow obstruction. They had mild symptoms, such as cough, sputum, and exertional breathlessness, without need for therapy. All subjects had no symptoms or history of asthma or allergic diseases.

**Table 3:** Subjects’ characteristics for histochemical analysis

	**Non-smokers**	**Smokers without COPD**	**COPD**
Number of subjects (M / F)	7 (2 / 5)	7 (6 / 1)	6 (6 / 0)
Age (yrs)	57.0 ± 2.5	65.1 ± 3.7	65.7 ± 4.1
Current / ex-smokers	-	4 / 3	0 / 6
Brinkman index (pack·years)	-	53.0 ± 6.9	69.2 ± 12.3
VC (%predicted)	107.9 ± 3.8	95.6 ± 3.9 *	94.3 ± 2.1 *
FEV_1_ (L)	2.4 ± 0.1	2.4 ± 0.3	2.1 ± 0.1
FEV_1_ / FVC (%)	83.3 ± 1.5	78.9 ± 2.7	65.7 ± 1.8 **††
FEV_1_ (%predicted)	111.1 ± 2.6	101.3 ± 4.1 *	83.5 ± 1.6 **††
PaO_2_ (mmHg)	83.0 ± 1.6	76.2 ± 2.9	73.6 ± 4.6
PaCO_2_ (mmHg)	39.1 ± 1.4	41.0 ± 1.1	39.3 ± 1.3
Type of lung cancers (number of subjects)	adenocarcinoma (5), AAH (1), metastasis of colon cancer (1),	adenocarcinoma (4), SCC (3)	adenocarcinoma (1), SCC (2), metastasis of colon cancer (1), LCC (1), LCNEC (1)

Results are means ± SEMs. Comparisons between groups were made by Student's t-tests (* p ≤ 0.05, ** p ≤ 0.01, compared with non-smokers; †† p ≤ 0.01, compared with smokers without COPD.). AAH; atypical adenomatous hyperplasia, SCC; squamous cell carcinoma, LCC; large cell carcinoma, LCNEC; large cell neuroendocrine carcinoma.

For immunohistochemical analyses, an anti-CLCA1 antibody was produced in rabbits as previously described [12]. Anti-MUC5AC antibody was from LabVision Corporation (Fremont, CA) and anti-IL-13Rα antibody was from Techne Corporation (Minneapolis, MN). Frozen tissue sections were fixed with 4% paraformaldehyde and incubated with antibodies. The secondary antibody used was ENVISION^+^ peroxidase (Dako, Glostrup, Denmark). Color was developed after incubation with DAB^+^ (Dako). Periodic acid Schiff (PAS) staining was used for mucus detection. Stained sections were counterstained with hematoxylin. For each patient specimen, airway epithelial areas were determined in a blinded manner for 6 randomly selected sites of well-preserved airway epithelium. Computer-assisted quantification of the staining in the selected areas was performed using MacSCOPE (Mitani Corporation, Fukui, Japan).

### Statistical Analysis

Results are expressed as means ± SEMs. Comparisons between groups were performed using Student's *t*-test for paired or unpaired data with Holm’s corrections. Associations between variables were assessed by Pearson correlation coefficients. P-values ≤ 0.05 were considered significant. Statistical analyses used SAS System Version 8.2 (SAS Institute Inc., Cary, NC).

## Results

### Quantitative analysis of CLCA1 mRNA in induced-sputum cells

**Figure 1:**
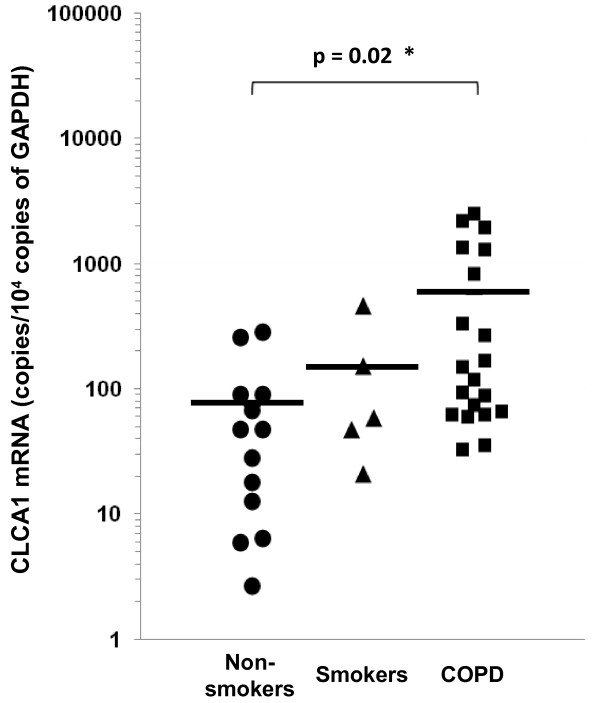
**Real-time quantitative PCR analyses of CLCA1 mRNA expression in induced sputum cells.** Induced sputum samples were obtained from non-smokers (circles, n = 13), smokers without COPD (triangles, n = 5), and COPD patients (squares, n = 20), as described in Methods. Group mean values are shown by horizontal lines. CLCA1 mRNA expression (normalized to that of GAPDH mRNA) was significantly greater for COPD patients’ cells than for non-smokers’ cells. * p ≤ 0.05, Student’s *t*-test.

CLCA1 mRNA expression levels were determined in the induced-sputum cells of 20 COPD patients, 5 smokers without COPD, and 13 healthy non-smokers. Table 2 shows the differential characteristics of the inflammatory cells observed in these sputum samples. Compared with samples from non-smokers, there were increased proportions of neutrophils (82.5 ± 4.0%, p = 0.009) and eosinophils (5.5 ± 2.0%, p = 0.05) in COPD patient samples, which was consistent with COPD. Smokers also showed a tendency for an increased proportion of neutrophils (73.8 ± 11.8%). In addition, COPD patient samples had a significantly decreased proportion of macrophages (8.3 ± 2.8%, p = 0.0004). This indicated that the origin of sputum from COPD patients was relatively close to the central airways, as macrophages are generally located in alveolar areas or the peripheral airways. Using real-time quantitative PCR analyses, there was a significant increase in the expression of CLCA1 mRNA per 10,000 copies of GAPDH mRNA in sputum cells from COPD patients (579 ± 179 copies, p = 0.02) compared to cells from non-smokers (73 ± 26 copies) (Figure 1). No significant difference was found between smokers (148 ± 81 copies) and non-smokers, even though there was a tendency for increased CLCA1 mRNA expression in smokers. In addition, there was a significant positive correlation between CLCA1 mRNA expression and the proportions of neutrophils (r = 0.34, p = 0.04). There were no significant correlations with other inflammatory cells (results not shown). Because neutrophil infiltration is a major pathological feature of COPD, the observed positive correlation suggested that CLCA1 expression was induced during the course of COPD development.

### CLCA1 protein expression in airway epithelia

**Figure 2:**
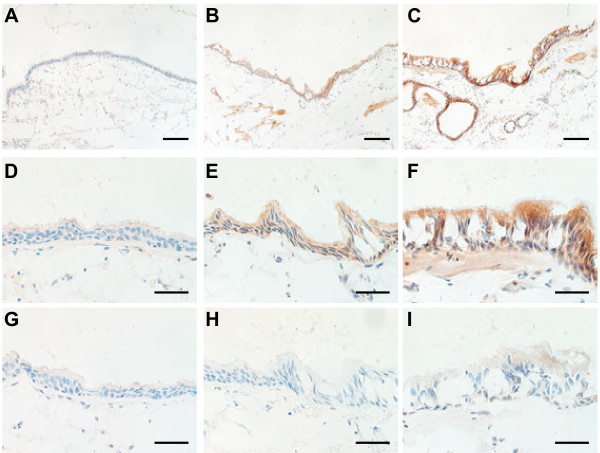
**CLCA1 protein expression in airway epithelium.** Immunohistochemical staining for CLCA1 in lung sections from (A) a non-smoker, (B) a smoker without COPD, and (C) a COPD patient. (**A-C**) Scale bars = 100 μm. CLCA1 protein was strongly expressed in the airway epithelia (bronchioles and terminal bronchioles) and minimally expressed in smooth muscles of the smoker and COPD patient. (**D-F**) Magnified images of panel A (D), panel B (E), and panel C (F). (**G-I**) Control experiments using rabbit IgG for the same lung sections from (G) a non-smoker, (H) a smoker without COPD, and (I) a COPD patient (D-I: scale bars = 40 μm).

**Figure 3:**
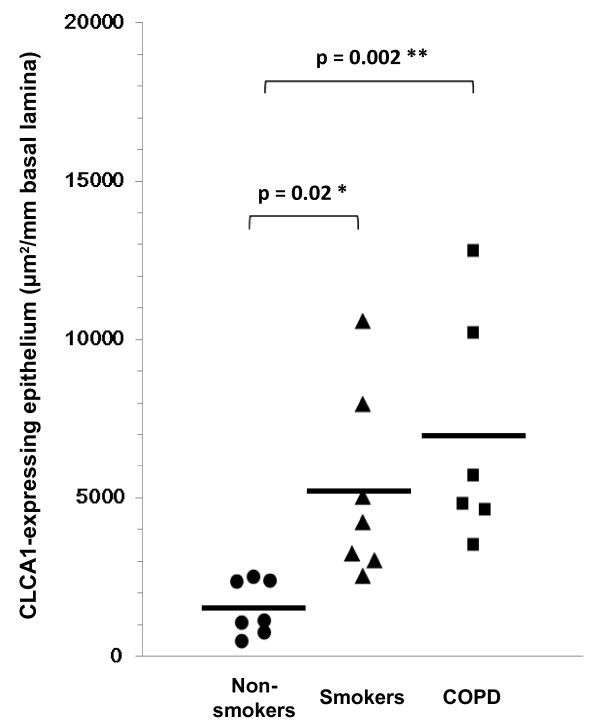
**Semi-quantitative image analyses of airway epithelium.** Total CLCA1-expressing areas of epithelia were determined with a computerized image analysis system as described in Methods for non-smokers (circles, n = 7), smokers without COPD (triangles, n = 7), and COPD patients (squares, n = 6). Group mean values are shown by horizontal lines. * p ≤ 0.05, ** p ≤ 0.01, Student’s *t*-test.

Using immunohistochemical staining with an anti-CLCA1 antibody, we compared CLCA1 protein expression in lung tissue sections from non-smokers, smokers without COPD, and COPD patients (Figure 2). For smokers (Figure 2B, 2E) and COPD patients (Figure 2C, 2F), there was prominent CLCA1 protein expression in the airway epithelia (bronchioles and terminal bronchioles) and minimal expression in smooth muscles. In contrast, few CLCA1-expressing cells were detected in the lung sections from non-smokers (Figure 2A, 2D). To evaluate relationships between CLCA1 protein expression and COPD clinical characteristics, we performed semi-quantitative image analyses of stained sections from 6 COPD patients, 7 smokers without COPD, and 7 non-smokers. For each sample, the total areas of airway epithelia were measured for a minimum of 6 different sites of well-preserved airway epithelium; these results were expressed as units per millimeter of basal lamina. The average lengths of basal lamina per unit area were 2.4 ± 0.1 mm in non-smokers, 2.5 ± 0.4 mm in smokers, and 2.5 ± 0.1 mm in COPD patients, which established that these comparisons were made among airway epithelia of similar sizes. The average area of CLCA1-expressing epithelium per millimeter of basal lamina was significantly greater for smokers (5232 ± 1126 μm^2^, p = 0.02) and COPD patients (6954 ± 1504 μm^2^, p = 0.002) than for non-smokers (1524 ± 326 μm^2^) (Figure 3). No significant difference was found between smokers and COPD patients.

### Relationships between CLCA1 protein expression and lung function parameters

**Figure 4:**
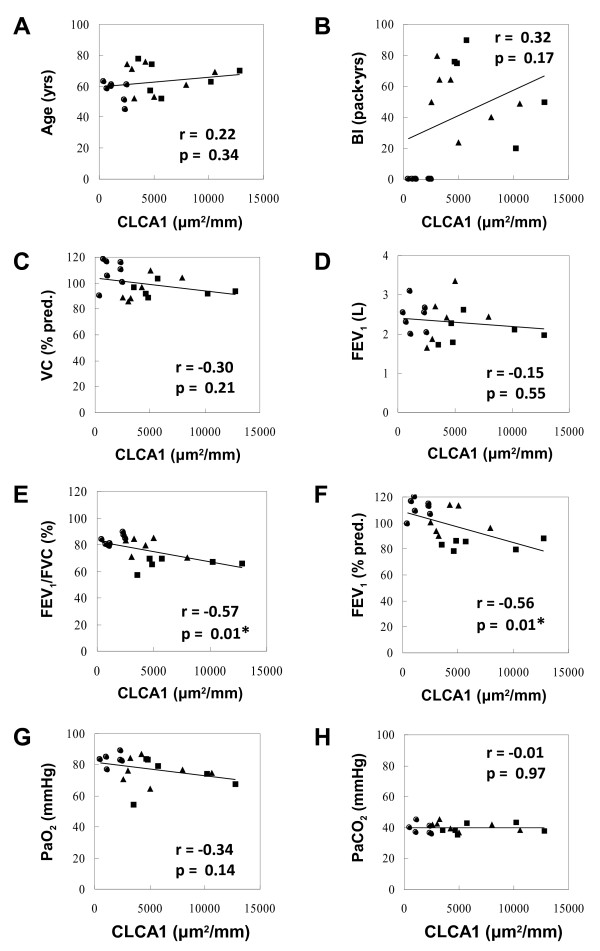
**Correlations between lung function parameters and CLCA1-expressing epithelia.** (**A**) Age and CLCA1 protein expression. (**B**) Brinkman index and CLCA1. (**C**) %predicted VC and CLCA1. (**D**) FEV_1_ and CLCA1. (**E**) FEV_1_/FVC and CLCA1. (**F**) %predicted FEV_1_ and CLCA1. (**G**) PaO_2_ and CLCA1. (**H**) PaCO_2_ and CLCA1. Results are for non-smokers (circles, n = 7), smokers without COPD (triangles, n = 7), and COPD patients (squares, n = 6). Associations between variables were assessed by Pearson correlation coefficients (* p ≤ 0.05).

We also assessed the relationships between CLCA1 protein expression image data and different clinical parameters shown in Table 3 (Figure 4). There were significant negative correlations between CLCA1 protein expression with FEV_1_/FVC (r = −0.57, p = 0.01, Figure 4E) and %predicted FEV_1_ (r = −0.56, p = 0.01, Figure 4F). In contrast, other clinical variables, including age, Brinkman index (BI), VC, FEV_1_, PaO_2_, and PaCO_2_, did not show significant correlations with CLCA1 protein expression in airway epithelia.

### Histochemical comparison of CLCA1 protein expression and mucus production

**Figure 5:**
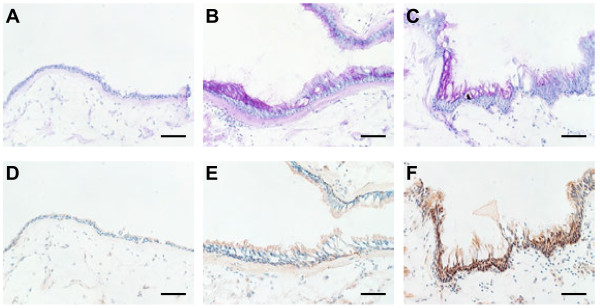
**Coincidence of PAS-stained areas and CLCA1-expressing cells in airway epithelium.** Lung sections were stained with PAS (A-C) or immunohistochemically stained with anti-CLCA1 antibody (D-F). (**A**, **D**) Airway epithelium of a non-smoker. Few mucus and CLCA1-expressing cells were detected. (**B**, **E**) Airway epithelium of a smoker without COPD. Increased numbers of mucus secretory cells, mostly goblet cells, were observed. (**C**, **F**) Airway epithelium of a COPD patient. Significant coincidence between mucus overproduction and CLCA1 expression was observed. Scale bars = 50 μm.

**Figure 6:**
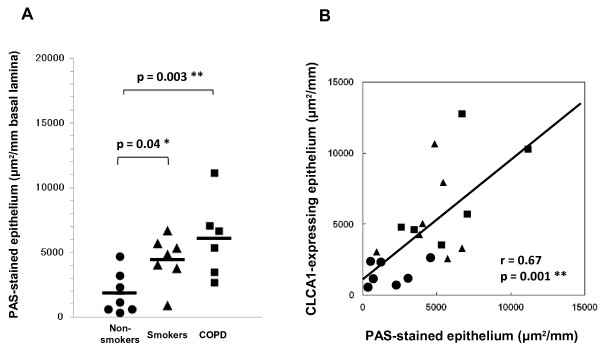
**Semi-quantitative image analyses of PAS-stained epithelium.** (**A**) The epithelial areas of interest for non-smokers (circles, n = 7), smokers without COPD (triangles, n = 7), and COPD patients (squares, n = 6) were determined with a computerized image analysis system, as described in Methods. Group mean values are shown by horizontal lines. * p ≤ 0.05, ** p ≤ 0.01, Student’s *t*-test. (**B**) Correlation between CLCA1 expression and PAS-stained areas in airway epithelium (Pearson’s r = 0.67, ** p ≤ 0.01).

To evaluate the relationship between CLCA1 protein expression and mucus production in the airway epithelium, we used PAS staining and CLCA1 immunostaining for serial lung sections (Figure 5) and quantified the image data for these stained sections. The average area of PAS-stained epithelium per millimeter of basal lamina was significantly larger for COPD patients (6042 ± 1241 μm^2^, p = 0.003) and smokers without COPD (4471 ± 706 μm^2^, p = 0.04) than for non-smokers (1823 ± 615 μm^2^) (Figure 6A). No significant difference was found between smokers and COPD patients, even though there was a tendency for increased mucus production in COPD patients. We also compared PAS-stained epithelium and CLCA1-expressing epithelium (Figure 6B). The area of PAS-stained epithelium was significantly positively correlated with the area of CLCA1-expressing epithelium (r = 0.67, p = 0.001). These results suggested that CLCA1 may play a critical role in mucus production in the airway epithelia of smokers and COPD patients, similar to what is observed in asthma.

### Relationship between CLCA1 expression and an IL-13 signal in COPD

**Figure 7:**
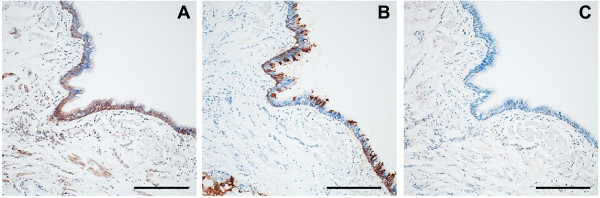
**Coincidence of CLCA1 expression and MUC5AC expression, but not IL-13 receptor expression.** Lung sections from a COPD patient were immunohistochemically stained with (**A**) anti-CLCA1 antibody, (**B**) anti-MUC5AC antibody, or (**C**) anti-IL-13Rα antibody. Scale bars = 200 μm.

In a previous report, the expression of CLCA1 and MUC5AC, one of the mucin proteins, was induced by stimulating human bronchial epithelial cells with IL-13 [14]. To examine a possible relationship between CLCA1 expression and an IL-13 signal in COPD, we immunostained lung sections from a COPD patient for MUC5AC and IL-13 receptor-alpha (IL-13Rα). MUC5AC protein was detected in airway epithelia, and MUC5AC expression mostly coincided with the expression of CLCA1 (Figure 7A, 7B), as previously reported in asthma [12]. In contrast, there was little expression of IL-13Rα protein in the airway epithelia of this COPD patient (Figure 7C), whereas IL-13Rα expression was observed in the airway epithelia of asthmatic patients [15]. These results suggest that CLCA1 expression in COPD may be induced via a pathway that is different from that in asthma.

## Discussion

We previously established that the expression of CLCA1 was associated with asthma pathology [11,12]. In this report, we investigated the possibility that CLCA1 expression was associated with COPD pathology, characterized by irreversible airflow obstruction and chronic mucus hypersecretion. CLCA1 mRNA expression in induced sputum cells tended to increase with smoking history, and there was a significant increase in these cells from COPD patients. Immunohistochemical analyses using frozen lung tissue sections from non-smokers, smokers without COPD, and COPD patients showed that CLCA1 protein expression was increased in the lung tissues of smokers and COPD patients. These results suggest that CLCA1 protein expression is gradually up-regulated due to smoking.

Wang et al. reported that the expression of CLCA1 mRNA, but not protein, was increased in the airways of Chinese patients with COPD [16]. In their study, they examined these expressions in non-smokers and former smokers who had given up smoking for more than 5 years, but did not examine the direct influence of cigarette smoking. Hauber et al. also observed increased expression of CLCA1 in the airways of patients with obstructive chronic bronchitis, but they did not discuss the relationship between CLCA1 and smoking [17]. It has been shown that cigarette smoke exposure induced the expression of CLCA1 mRNA in the trachea and lung tissues of rats [18]. Our results also suggest the importance of cigarette smoking for CLCA1 expression in the airway for those with a smoking history and for COPD patients. Furthermore, we identified significant negative correlations between CLCA1 expression and lung function results, including FEV_1_/FVC and %predicted FEV_1_. CLCA1 protein expression was gradually up-regulated due to smoking and may be associated in some way with impaired lung function. This is the first study to demonstrate a critical relationship between CLCA1 expression, smoking, and COPD.

Airway mucus obstruction is a common feature of COPD, asthma, and many respiratory diseases [19-22]. CLCA1 is one of the key molecules associated with mucus production and secretion by airway epithelial cells in bronchial asthma. CLCA1 is found in bronchial epithelium, particularly in mucus-producing goblet cells, and its protein expression levels are highly correlated with the levels of MUC5AC expression and PAS staining in asthmatics’ tissues [12]. In this study, we found good correlations between PAS-stained areas, CLCA1-expressing areas, and MUC5AC-expressing areas in the airway epithelia of smokers and COPD patients. COPD pathology is specifically associated with an increased expression of MUC5AC in the bronchiolar epithelium [23]. Our results suggest that CLCA1 plays a critical role in COPD by inducing mucus production in the airway epithelia, similar to what is observed in asthma.

In this study, we identified that cigarette smoking was implicated in the pathophysiology of COPD by inducing mucus overproduction via CLCA1 expression. However, the mechanism by which smoking induces CLCA1 expression remains poorly understood. In asthma, IL-13 is a key molecule that links CLCA1 expression with mucus production. IL-13 stimulation can induce CLCA1 and MUC5AC expression in normal human bronchial epithelial cells and murine asthma models [14,24]. However, when we examined for the expression of IL-13Rα protein in the airway epithelia of COPD patients, which would suggest the presence of an IL-13 signaling pathway, we were unable to detect IL-13Rα protein expression. Although both COPD and asthma are associated with chronic inflammation of the respiratory tract, there are marked differences in the inflammatory cells and mediators involved in these two diseases [19]. COPD pathology is mainly associated with neutrophils, macrophages, CD8^+^ T cells, Th1 cytokines, and IL-8, whereas asthma pathology is associated with eosinophils, macrophages, Th2 cytokines, and eotaxin. Although the participation of IL-13 and other Th2 cytokines in COPD, including chronic bronchitis, has also been reported in several studies [25,26], they may be not important contributors to CLCA1 expression in COPD.

Multiple stimuli, including oxidants in cigarette smoke and cytokines produced by inflammatory cells, may induce CLCA1 expression and enhance mucus hypersecretion. It has been reported that chronic administration of TNF-α, one of the cytokines induced by cigarette smoke, increased the expression of mCLCA3 (mouse counterpart of CLCA1) and MUC5AC mRNA in mouse lung tissues [27]. TNF-α enhances the expression of epidermal growth factor receptor (EGF-R), which co-localizes with MUC-5 AC protein [28]. Hegab et al. found that cigarette smoke induced the expression of EGF-R mRNA, as well as those of rCLCA1 and MUC5AC [18]. They also reported that a selective EGF-R tyrosine kinase inhibitor, AG-1478, and a CLCA1 inhibitor, niflumic acid, blocked EGF-induced MUC5AC expression and mucus staining in the human airway epithelial cell line NCI-H292. These studies suggest that cigarette smoke activation of EGF-R signaling may be involved in CLCA1 expression and mucus overproduction.

IL-17 induced by cigarette smoke may also be one of the key molecules that connect CLCA1 expression, mucus production, smoking, and COPD. Hashimoto et al. reported that *STAT1 KO* mice infected with respiratory syncytial virus (RSV) had upregulated mCLCA3 and MUC5AC expressions and IL-17 production in the lungs [29]. IL-17 is expressed by macrophages, neutrophils, CD4^+^, and CD8^+^ T cells in the airways of COPD patients and can play important roles in COPD pathogenesis [30,31]. Moreover, the overexpression of IL-17F in murine lung epithelium results in lymphocyte and macrophage infiltration and mucus hyperplasia [32]. These studies suggest that IL-17 induced by cigarette smoke may lead to CLCA1 expression and mucus hyperplasia, which can exacerbate COPD. However, in this study we could not establish these hypotheses. Additional studies will be needed to identify the mechanism(s) responsible for mucus induction via CLCA1 expression in COPD patients.

## Conclusions

Taken together, CLCA1 expression was increased in accordance with smoking history and was significantly increased in the lungs of COPD patients. CLCA1 expression was significantly correlated with neutrophil infiltration, respiratory disability, and mucus production in airway epithelia. CLCA1 may contribute to the development and pathogenesis of COPD by inducing mucus production. Targeting CLCA1 may prove to be an effective therapeutic approach for treating COPD.

## Abbreviations

CLCA: Ca^2+^-activated Cl^-^ channel; COPD: Chronic obstructive pulmonary disease; FEV_1_: Forced expiratory volume in 1 second; FVC: Forced vital capacity; PAS: Periodic acid Schiff; BI: Brinkman index; VC: Vital capacity; PaO_2_: Arterial partial pressure of oxygen; PaCO_2_: Arterial partial pressure of carbon dioxide.

## Competing interests

The authors declare that they have no competing interests.

## Authors’ contributions

HI did the immunohistochemical studies, performed the statistical analysis, and drafted the original manuscript. KF supervised the clinical characterizations, coordinated sputum analysis, and drafted the original manuscript. SM contributed to conceiving the project design, and performed the molecular genetic studies. AN contributed to conceiving the project design, and helped to draft the manuscript. KK contributed to conceiving the project design, and approved clinical studies for this project. All authors read and approved the final manuscript.

## References

[B1] Pauwels RA Buist AS Calverley PM Jenkins CR Hurd SS Global strategy for the diagnosis, management, and prevention of chronic obstructive pulmonary disease: NHLBI/WHO global initiative for chronic obstructive lung disease (GOLD) workshop summary Am J Respir Crit Care Med 2001 163 1256 1276 11316667 10.1164/ajrccm.163.5.2101039

[B2] Snider GL Chronic obstructive pulmonary disease: risk factors, pathophysiology and pathogenesis Ann Rev Med 1989 40 411 429 10.1146/annurev.me.40.020189.002211 2658758

[B3] MacNee W Oxidants/antioxidants and COPD Chest 2000 117 303S 317S 10.1378/chest.117.5_suppl_1.303S 10843965

[B4] Maestrelli P Saetta M Mapp CE Fabbri LM Remodeling in response to infection and injury Am J Respir Crit Care Med 2001 164 S76 S80 11734472 10.1164/ajrccm.164.supplement_2.2106067

[B5] Shimura S Signal transduction of mucous secretion by bronchial gland cells Cell Signal 2000 12 271 277 10.1016/S0898-6568(00)00066-8 10822167

[B6] Clarke LL Grubb BR Gabriel SE Smithies O Koller BH Boucher RC Defective epithelial chloride transport in a gene-targeted mouse model of cystic fibrosis Science 1992 257 1125 1128 10.1126/science.257.5073.1125 1380724

[B7] Clarke LL Grubb BR Yankaskas JR Cotton CU McKenzie A Boucher RC Relationship of a non-cystic fibrosis transmembrane conductance regulator-mediated chloride conductance to organ-level disease in Cftr(−/−) mice Proc Natl Acad Sci U S A 1994 91 479 483 10.1073/pnas.91.2.479 7507247 PMC42972

[B8] Knowles MR Clarke LL Boucher RC Activation by extracellular nucleotides of chloride secretion in the airway epithelia of patients with cystic fibrosis N Engl J Med 1991 325 533 538 10.1056/NEJM199108223250802 1857389

[B9] Loewen ME Forsyth GW Structure and function of CLCA proteins Physiol Rev 2005 85 1061 1092 10.1152/physrev.00016.2004 15987802

[B10] Agnel M Vermat T Culouscou J-M Identification of three novel members of the calcium-dependent chloride channel (CaCC) family predominantly expressed in the digestive tract and trachea FEBS Lett 1999 455 295 301 10.1016/S0014-5793(99)00891-1 10437792

[B11] Nakanishi A Morita S Iwashita H Sagiya Y Ashida Y Shirafuji H Fujisawa Y Nishimura O Fujino M Role of gob-5 in mucus overproduction and airway hyperresponsiveness in asthma Proc Natl Acad Sci U S A 2001 98 5175 5180 10.1073/pnas.081510898 11296262 PMC33183

[B12] Hoshino M Morita S Iwashita H Sagiya Y Nagi T Nakanishi A Ashida Y Nishimura O Fujisawa Y Fujino M Increased expression of the human Ca2+-activated chloride channel 1 (CaCC1) gene in asthmatic airway Am J Respir Crit Care Med 2002 165 1132 1136 11956057 10.1164/ajrccm.165.8.2107068

[B13] Fujimoto K Kubo K Yamamoto H Yamaguchi S Matsuzawa Y Eosinophilic inflammation in the airway is related to glucocorticoid reversibility in patients with pulmonary emphysema Chest 1999 115 697 702 10.1378/chest.115.3.697 10084478

[B14] Yasuo M Fujimoto K Tanabe T Yaegashi H Tsushima K Takasuna K Koike T Yamaya M Nikaido T Relationship between calcium-activated chloride channel 1 and MUC5AC in goblet cell hyperplasia induced by interleukin-13 in human bronchial epithelial cells Respiration 2006 73 347 359 10.1159/000091391 16465045

[B15] Heinzmann A Mao XQ Akaiwa M Kreomer RT Gao PS Ohshima K Umeshita R Abe Y Braun S Yamashita T Roberts MH Sugimoto R Arima K Arinobu Y Yu B Kruse S Enomoto T Dake Y Kawai M Shimazu S Sasaki S Adra CN Kitaichi M Inoue H Yamauchi K Tomichi N Kurimoto F Hamasaki N Hopkin JM Izuhara K Shirakawa T Deichmann KA Genetic variants of IL-13 signaling and human asthma and atopy Hum Mol Gen 2000 9 549 559 10.1093/hmg/9.4.549 10699178

[B16] Wang K Feng YL Wen FQ Chen XR Ou XM Xu D Yang J Deng ZP Increased expression of human calcium-activated chloride channel 1 is correlated with mucus overproduction in the airways of Chinese patients with chronic obstructive pulmonary disease Chin Med J 2007 120 1051 1057 17637221

[B17] Hauber H-P Bergeron C Tsicopoulos A Wallaert B Olivenstein R Holroyd KJ Levitt RC Hamid Q Increased expression of the calcium-activated chloride channel hCLCA1 in airways of patients with obstructive chronic bronchitis Can Respir J 2005 12 143 146 15875066 10.1155/2005/531432

[B18] Hegab AE Sakamoto T Nomura A Ishii Y Morishima Y Iizuka T Kiwamoto T Matsuno Y Homma S Sekizawa K Niflumic acid and AG-1478 reduce cigarette smoke-induced mucin synthesis: the role of hCLCA1 Chest 2007 131 1149 1156 10.1378/chest.06-2031 17426222

[B19] Jeffery PK Comparison of the structural and inflammatory features of COPD and asthma Chest 2000 117 251S 260S 10.1378/chest.117.5_suppl_1.251S 10843939

[B20] Rogers DF Mucus pathophysiology in COPD Monaldi Arch Chest Dis 2000 55 324 332 11057087

[B21] Rogers DF Airway goblet cells: responsive and adaptable front-line defenders Eur Respir J 1994 7 1690 1706 10.1183/09031936.94.07091678 7995400

[B22] Rogers DF The airway goblet cell Int J Biochem Cell Biol 2003 35 1 6 10.1016/S1357-2725(02)00083-3 12467641

[B23] Caramori G Di Gregorio C Carlstedt I Casolari P Guzzinati I Adcock IM Barnes PJ Ciaccia A Cavallesco G Chung KF Papi A Mucin expression in peripheral airways of patients with chronic obstructive pulmonary disease Histopathology 2004 45 477 484 10.1111/j.1365-2559.2004.01952.x 15500651

[B24] Nakano T Inoue H Fukuyama S Matsumoto K Matsumura M Tsuda M Matsumoto T Aizawa H Nakanishi Y Niflumic acid suppresses interleukin-13-induced asthma phenotypes Am J Respir Crit Care Med 2006 173 1216 1221 10.1164/rccm.200410-1420OC 16528019

[B25] Miotto D Ruggieri MP Boschetto P Cavallesco G Papi A Bononi I Piola C Murer B Fabbri LM Mapp CE Interleukin-13 and −4 expression in the central airways of smokers with chronic bronchitis Eur Respir J 2003 22 602 608 10.1183/09031936.03.00046402 14582911

[B26] Zhu J Majumdar S Qiu Y Ansari T Oliva A Kips JC Pauwels RA De Rose V Jeffery PK Interleukin-4 and interleukin-5 gene expression and inflammation in the mucus-secreting glands and subepithelial tissue of smokers with chronic bronchitis Am J Respir Crit Care Med 2001 164 2220 2228 11751191 10.1164/ajrccm.164.12.2009060

[B27] Busse PJ Zhang TF Srivastava K Lin BP Schofield B Sealfon SC Li X-M Chronic exposure to TNF-alpha increases airway mucus gene expression in vivo J Allergy Clin Immunol 2005 116 1256 1263 10.1016/j.jaci.2005.08.059 16337454

[B28] Takeyama K Jung B Shim JJ Burgel PR Dao-Pick T Ueki IF Protin U Kroschel P Nadel JA Activation of epidermal growth factor receptors is responsible for mucin synthesis induced by cigarette smoke Am J Physiol Lung Cell Mol Physiol 2001 280 L165 L172 11133506 10.1152/ajplung.2001.280.1.L165

[B29] Hashimoto K Durbin JE Zhou W Collins RD Ho SB Kolls JK Dubin PJ Sheller JR Goleniewska K O'Neal JF Olson SJ Mitchell D Graham BS Peebles RS Respiratory syncytial virus infection in the absence of STAT 1 results in airway dysfunction, airway mucus, and augmented IL-17 levels J Allergy Clin Immunol 2005 116 550 557 10.1016/j.jaci.2005.03.051 16159623

[B30] Eustace A Smyth LJ Mitchell L Williamson K Plumb J Singh D Identification of cells expressing IL-17A and IL-17 F in the lungs of patients with COPD Chest 2011 139 1089 1100 10.1378/chest.10-0779 20864612

[B31] Chang Y Nadigel J Boulais N Bourbeau J Maltais F Eidelman DH Hamid Q CD8 positive T cells express IL-17 in patients with chronic obstructive pulmonary disease Respir Res 2011 12 43 52 10.1186/1465-9921-12-43 21477350 PMC3082241

[B32] Yang XO Chang SH Park H Nurieva R Shah B Acero L Wang YH Schluns KS Broaddus RR Zhu Z Dong C Regulation of inflammatory responses by IL-17 F J Exp Med 2008 205 1063 1075 10.1084/jem.20071978 18411338 PMC2373839

